# Estimating true evolutionary distances under rearrangements, duplications, and losses

**DOI:** 10.1186/1471-2105-11-S1-S54

**Published:** 2010-01-18

**Authors:** Yu Lin, Vaibhav Rajan, Krister M Swenson, Bernard ME Moret

**Affiliations:** 1 Laboratory for Computational Biology and Bioinformatics, Swiss Federal Institute of Technology (EPFL), EPFL-IIS-LCBB, INJ 230, Station 14, CH-1015 Lausanne, Switzerland

## Abstract

**Background:**

The rapidly increasing availability of whole-genome sequences has enabled the study of whole-genome evolution. Evolutionary mechanisms based on genome rearrangements have attracted much attention and given rise to many models; somewhat independently, the mechanisms of gene duplication and loss have seen much work. However, the two are not independent and thus require a unified treatment, which remains missing to date. Moreover, existing rearrangement models do not fit the dichotomy between most prokaryotic genomes (one circular chromosome) and most eukaryotic genomes (multiple linear chromosomes).

**Results:**

To handle rearrangements, gene duplications and losses, we propose a new evolutionary model and the corresponding method for estimating true evolutionary distance. Our model, inspired from the DCJ model, is simple and the first to respect the prokaryotic/eukaryotic structural dichotomy. Experimental results on a wide variety of genome structures demonstrate the very high accuracy and robustness of our distance estimator.

**Conclusion:**

We give the first robust, statistically based, estimate of genomic pairwise distances based on rearrangements, duplications and losses, under a model that respects the structural dichotomy between prokaryotic and eukaryotic genomes. Accurate and robust estimates in true evolutionary distances should translate into much better phylogenetic reconstructions as well as more accurate genomic alignments, while our new model of genome rearrangements provides another refinement in simplicity and verisimilitude.

## Background

### Introduction

Interest in the evolution of genome structure has been growing steadily in the last 10 years, sustained in part by the ever increasing number of sequenced genomes. In particular much work has been done on rearrangements (see, e.g., [1]), using the convention that each chromosome of the genome is represented by an ordered list of identifiers, each identifier referring to a syntenic block or, more commonly, to a member of a gene family. (In the following, we shall use the word "gene" in a broad sense to denote elements of such orderings and refer to such orderings as "gene orders".) Variations in the placement of homologous genes, as well as variations in gene content and multiplicity, among organisms can then be analyzed. Such data is of great interest to evolutionary biologists, but also to comparative genomicists and to any researcher interested in understanding evolutionary changes in pathogens, crop plants, and, more generally, the biome.

The most fundamental task in the analysis of such data is to estimate the amount of evolutionary change between two genomes--that is, to compute a pairwise *evolutionary distance*. The *true distance*, that is, the number of *actual *evolutionary events (rearrangements, duplications, and losses) that took place during the course of evolution, is what we want to obtain, but is not, of course, something that we can compute. Researchers have thus used a two-stage process, in which a well defined measure is first computed (such as an *edit distance*, that is, the *smallest *number of evolutionary events needed to transform one genome into the other), then a statistical model of evolution is used to infer an estimate of the true distance by deriving the effect of a given number of changes in the model on the computed measure and (algebraically or numerically) inverting the derivation to produce a maximum-likelihood estimate of the true distance under the model. This second step is usually called a *distance correction *and has long been used for sequence (DNA) data (see, e.g., [2]) as well as, more recently, for gene-order data, for which see [3-7].

Evolutionary events that affect the gene order of genomes include various rearrangements, which affect only the order, and gene duplications and losses, which affect both the gene content and, indirectly, the order. (Gene insertion, corresponding to lateral gene transfer or neofunctionalization, can be viewed as a special case of duplication.) Rearrangements themselves include inversion, transposition, block exchange, circularization and linearization, all of which act on a single chromosome, and translocation, fusion, and fission, which act on two chromosomes. All of these operations are subsumed in the *double*-*cut*-*and*-*join (DCJ) *[8,9], which has formed the basis for much of the algorithmic research on rearrangements over the last few years, including a statistically based method to estimate the true evolutionary distance between two genomes [7]. DCJ makes two cuts, which can be in the same chromosome or in two different chromosomes, producing four cut ends, then rejoins the four cut ends in any of the three possible ways. The DCJ model, however, is unrealistic in two major respects. First, if the two cuts are in the same chromosome, one of the two nontrivial rejoinings causes a fission, creating a new circular chromosome. However, circular chromosomes do not normally arise in organisms with linear chromosomes, and prokaryotic genomes normally consist of a single circular chromosome. Nor can this form of rejoining be forbidden as, without it, DCJ simply reduces to inversion. Secondly, DCJ is a model of rearrangements: it does not take into account evolutionary events that alter the gene content, such as duplications and losses.

Of these two problems, the first has not been seriously addressed: the model we present here is, to the best of our knowledge, the first model that naturally preserves the dichotomy between prokaryotic and eukaryotic genomes. While gene (or segment) duplications and losses have long been studied by geneticists and molecular biologist, their integration with rearrangements in a unified model has seen relatively little work to date. El-Mabrouk [10] gave an exact algorithm to compute edit distances for inversions and losses and also a heuristic to approximate edit distances for inversions, losses, and nonduplicating insertions (all of her results assume that genes cannot be duplicated). More recently, Yancopoulos and Friedberg [11] gave an algorithm to compute edit distances under deletions, insertions, duplications, and DCJ operations, under the constraint that each deletion can only remove a single gene. These and other approaches targeted the edit distance, not the true evolutionary distance. Swenson *et al*. [12] gave an algorithm to approximate the true evolutionary distance under deletions, insertions, duplications, and inversions for unichromosomal genomes and showed good results under simulations and for small-scale phylogenetic reconstruction. Rearrangements, duplications and losses have also been addressed in the framework of ancestral reconstruction (see, e.g., [13]). All of these approaches have focused on parsimony criteria and have used pre-assigned weights for the various operations.

In this paper, we propose a new evolutionary model which respects the dichotomy between prokaryotic and eukaryotic genomes and which takes gene duplications and losses into account. Using this new evolutionary model, we develop a statistically based method to estimate the true evolutionary distance in terms of the actual number of rearrangements, gene duplications, and gene losses. Finally, we provide extensive experimental results on a wide variety of genome structures to illustrate the robustness and high accuracy of our estimator.

### Genomes as gene-order data

**Figure 1:**
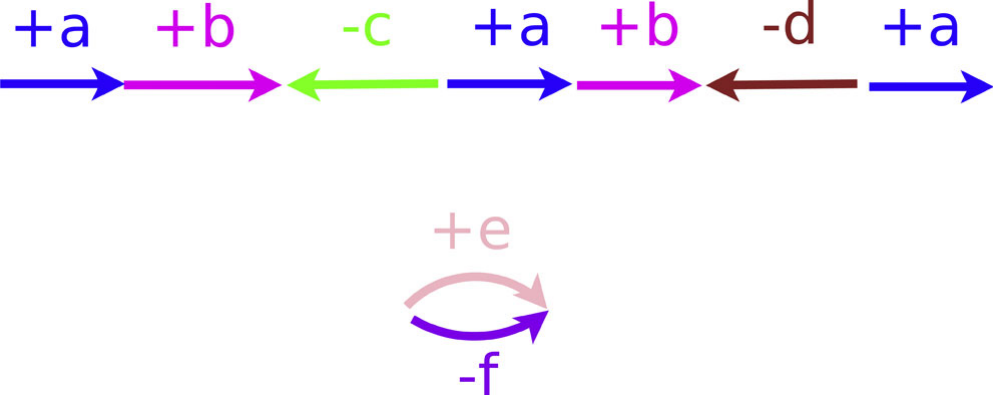
**A very small genome *G***.

We denote the tail of a gene *g *by *g*^*t *^and its head by *g*^*h*^. We write +*g *to indicate an orientation from tail to head (*g*^*t *^→ *g*^*h*^), -*g *otherwise (*g*^*h *^→ *g*^*t*^). Two consecutive genes *a *and *b *can be connected by one *adjacency *of one of the following four types: {*a*^*t*^, *b*^*t*^}, {*a*^*h*^, *b*^*t*^}, {*a*^*t*^, *b*^*h*^}, and {*a*^*h*^, *b*^*h*^}. If gene *c *lies at one end of a linear chromosome, then we also have a singleton set, {*c*^*t*^} or {*c*^*h*^}, called a *telomere*. A *genome *can then be represented as a multiset of genes together with a multiset of adjacencies and telomeres. For example, the toy genome in Figure 1, composed of one linear chromosome, (+*a*, +*b*, -*c*, +*a*, +*b*, -*d*, +*a*), and one circular one, (+*e*, -*f*), can be represented by the multiset of genes {*a*, *a*, *a*, *b*, *b*, *c*, *d*, *e*, *f*} and the multiset of adjacencies and telomeres {{*a*^*t*^}, {*a*^*h*^, *b*^*t*^}, {*b*^*h*^, *c*^*h*^}, {*c*^*t*^, *a*^*t*^}, {*a*^*h*^, *b*^*t*^}, {*b*^*h*^, *d*^*h*^}, {*d*^*t*^, *a*^*t*^}, {*a*^*h*^}, {*e*^*h*^, *f*^*h*^}, {*e*^*t*^, *f*^*t*^}}. Because of the duplicated genes, there is no one-to-one correspondence between genomes and multisets of genes, adjacencies, and telomeres. For example, the genome composed of one linear chromosome, (+*a*, +*b*, -*d*, +*a*, +*b*, -*c*, +*a*) and one circular one (+*e*, -*f*) would have the same multisets of genes, adjacencies and telomeres as that in Figure 1.

### Preliminaries on the evolutionary model

We use two parameters: the probability of occurrence of a gene duplication, *p*_*d*_, and the probability of occurrence of a gene loss, *p*_*l*_; the probability of occurrence of a rearrangement is then just *p*_*r *_= 1 - *p*_*d *_- *p*_*l*_. The next event is chosen from the three categories according to these parameters.

For rearrangements, we will select two adjacencies or telomeres with replacement uniformly from the multiset of all adjacencies and telomeres and then decide which rearrangement event we apply. The four cases are as follows.

#### Select two different adjacencies, or one adjacency and one telomere, in the same chromosome

For example, select two different adjacencies  and  on one linear chromosome *C *= (*a*_1 _... *a*_*i*-1_*a*_*i *_... *a*_*j*_*a*_*j*+1 _... *a*_*n*_). Reversing all genes between *a*_*i *_and *a*_*j *_yields (*a*_1 _... *a*_*i*-1 _- *a*_*j *_... -*a*_*i*_*a*_*j*+1 _... *a*_*n*_). Two adjacencies,  and , are replaced by two others,  and . This operation causes an inversion.

#### Select two adjacencies or one adjacency and one telomere in two different chromosomes

For example, select two adjacencies,  from one linear chromosome *C *= (*a*_1 _... *a*_*i*_*a*_*i*+1 _... *a*_*n*_) and  from another linear chromosome *D *= (*b*_1 _... *b*_*j*_*b*_*j*+1 _... *b*_*n*_). Now exchange the two segments between these two chromosomes *C *and *D*. There are two possible outcomes, (*a*_1 _... *a*_*i*_*b*_*j*+1 _... *b*_*n*_) and (*b*_1 _... *b*_*j*_*a*_*i*+1 _... *a*_*n*_) or (*a*_1 _... *a*_*i *_- *b*_*j *_... -*b*_1_) and (-*b*_*n *_... -*b*_*j*+1_*a*_*i*+1 _... *a*_*n*_). Two adjacencies,  and , are replaced by  and  or  and . This operation causes a translocation (or, if at least one chromosome is circular, a fusion).

#### Select the same adjacency twice

For example, select the adjacency  twice from linear chromosome *C *= (*a*_1 _... *a*_*i*_*a*_*i*+1 _... *a*_*n*_). Then split *C *into two new linear chromosomes, (*a*_1 _... *a*_*i*_) and (*a*_*i*+1 _... *a*_*n*_). The adjacency  is replaced by two telomeres  and . This operation causes a fission for a linear chromosome, a linearization for a circular one.

#### Select two telomeres

(Selecting one telomere twice is assimilated to selecting both telomeres of the linear chromosome.) For example, select telomeres  and  from two different linear chromosomes. Then concatenate these two linear chromosomes into a single new chromosome. Two telomeres,  and , are replaced by two other telomeres,  and . This operation causes a fusion on two linear chromosomes or a circularization on one linear chromosome.

For gene duplication, we uniformly select a position to start duplicating a short segment of chromosomal material and place the new copy to a new position within the genome. We set *L*_*max *_as the maximum number of genes in the duplicated segment and assume that the number of genes in that segment is a uniform random number between 1 and *L*_*max*_. For example, select one segment *a*_*i*+1 _... *a*_*i*+*L *_to duplicate and insert the copy between one adjacency . Such an operation duplicates *L *genes and *L *- 1 adjacencies, removes one adjacency, and adds two new adjacencies; thus genes *a*_*i*+1_,..., *a*_*i*+*L*-1 _and *a*_*i*+*L *_are added to the multiset of genes, the adjacency  is removed, and *L *+ 1 new adjacencies, , are added.

For gene loss, we restrict deletions to genes with at least two copies in the genome and we delete one gene at a time. We uniformly select one gene from the set of all candidate genes and delete it. For example, if we delete gene *a*_*i *_in the chromosome (... *a*_*i*-1_*a*_*i*_*a*_*i*+1 _...), one copy of *a*_*i *_is removed from the multiset of genes, while two adjacencies,  and , are replaced by one adjacency, .

## Methods

### An overview of our technique for estimating the true evolutionary distance

The problem of estimating the true evolutionary distance is defined as follows:

**Input**: The original genome *G *and the final genome *F*.

**Output**: An estimate of the actual number of evolutionary events that took place in the evolutionary history to transform *G *into *F*.

Based on the multisets of genes and of adjacencies and telomeres of *G*, for any genome G* of N* genes and l* linear chromosomes, we can build the vector , where *C *is the upper bound for the number of copies of one gene,  (*i *= 1,..., *C*) is the number of genes with *exactly i *copies in the genome G*,  (*i *= 1,..., *C*) is the number of adjacencies with *exactly i *copies in G* that also appear in *G*, DA* is the number of adjacencies in G* that do not appear in *G*, ST * is the number of telomeres in G* that also appear in *G*, and DT* is the number of telomeres in G* that do not appear in *G*. We can write

Let *G*^*k *^be the genome obtained from *G *= *G*^0 ^by applying *k *randomly selected evolutionary operations--under our model, the (*i *+ 1)st evolutionary operation is selected from all possible rearrangements, gene duplications, and gene losses on genome *G*^*i *^according to the parameters *p*_*d *_and *p*_*l*_. We can compute the vector  to represent the genome *G*^*k *^with respect to *G*.

In the section, we show that, given *G*, we can also produce the *estimate * for the expected vector *E*(*V*_*G*_(*G*^*k*^)), for any integer *k *> 0. Our approach for estimating the true evolutionary distance is then to return the integer *k *that minimizes the 1-norm distance between  and *V*_*G*_(*F*).

### Estimation of the expected vector after some number of random evolutionary events

Given the original genome *G*, the complete vector for genome *G*^*k *^is defined as , where  is the number of genes with exactly *i *copies in the genome *G*^*k*^,  (shared adjacencies) is the number of adjacencies with exactly *i *copies in *G*^*k *^that also appear in *G*, *DA*^*k *^(distinct adjacencies) is the number of adjacencies in *G*^*k *^that do not appear in *G*, *ST *^*k *^(shared telomeres) is the number of telomeres in *G*^*k *^that also appear in *G*, and *DT *^*k *^(distinct telomeres) is the number of telomeres in *G*^*k*^that do not appear in *G*.

Assume the original genome *G *has *N *genes, where each gene has at most *C *= *O*(1) copies, and *l *linear chromosomes, with *l *= *O*(1). We thus ignore items  and  for (*i *>*C*). The initial vector *V*_*G*_(*G*^0^) is then , where  is the number of genes with exactly *i *copies,  is the number of adjacencies with exactly *i *copies, *DA*^0 ^= 0, *ST *^0 ^= 2*l*, and *DT*^0 ^= 0. We now show how to update this vector under rearrangements, gene duplications and gene losses, respectively.

#### Rearrangements

We select two adjacencies or telomeres uniformly with replacement, from the multiset of all adjacencies or telomeres.

**Theorem 1 ***Assume all genomes have O*(1) *linear chromosomes, each gene has at most C *= *O*(1) *copies, and **represents the current genome G*^*k *^*based on the original genome G. For conciseness, write **(the total number of genes) and l*^*k *^= (*ST*^*k *^+*DT*^*k*^)/2 *(the number of linear chromosomes). Then we can write the expected vector for G*^*k*+1 ^*after one rearrangement operation: **where we have*

**Proof **In our evolutionary model, each rearrangement operation replaces old adjacencies or telomeres with new ones. Obviously, any rearrangement operation will not change the gene content, so  (*i *= 1,2,..., *C*) will be the same.

We first ignore the adjacencies or telomeres in the original genome *G *created after a rearrangement event. Remember two adjacencies or telomeres are selected with replacement uniformly from the multiset of all adjacencies and telomeres, and the number of all adjacencies and telomeres for genome *G*^*k *^is (*N*^*k *^+*l*^*k*^). Consider the multi-set *A*_*i *_of  adjacencies with exactly *i *copies in *G*^*k *^that also appear in *G*. The probability that exactly one of the two selected adjacencies is in *A*_*i *_is , the probability that two different adjacencies from *A*_*i *_are selected is , the probability that equivalent adjacencies from *A*_*i *_at different sites are selected is , and the probability that some adjacency from *A*_*i *_is selected twice is . For the time-being we ignore adjacencies and telomeres in *G *that may be created incidental. With probability  the number of adjacencies with exactly *i *copies decreases by *i*, and with probability  the number of adjacencies with exactly *i *copies decreases by 2*i*. With probability  the number of adjacencies with exactly (*i *-1) copies increases by (*i *-1), with probability  the number of adjacencies with exactly (*i *- 1) copies increases by 2(*i *- 1), and with probability  the number of adjacencies with exactly (*i *-2) copies increases by (*i *-2). Considering *i *= 1,2,..., *C *and *C *= *O*(1), we have

Now, we show that the correction for ignoring incidental creation of adjacencies or telomeres in *G *after a rearrangement event is  for each item. Consider any adjacency (*a*, *b*) in *G*: we might recover it only if we select two adjacencies or telomeres containing two genes *a *and *b*. Since each gene has at most *C *copies in the genome, there are at most *C*^2 ^pairs of adjacencies or telomeres that may lead to recovery of the adjacency (*a*, *b*). So, with probability at most , one specific adjacency in *G *might be created by the rearrangement. Summing up all the *N *- *l *adjacencies in *G*, we see that the correction for ignoring the possible newly created adjacencies or telomeres in *G *is .

Similarly, we can get  and .

#### Gene duplication

We select uniformly at random an integer between 1 and *L*_*max *_(the maximum number of genes in the duplicated segment), then select uniformly at random a position in the genome where to start the duplication, then insert the copy at another position selected uniformly at random.

**Theorem 2 ***Assume all genomes have O*(1) *linear chromosomes, each gene has at most C *= *O*(1) *copies, no two duplicate genes or adjacencies are within the segment to be duplicated, and **represents the current genome G*^*k *^*based on the original genome G. For conciseness, write **(the total number of genes), l*^*k *^= (*ST*^*k *^+ *DT*^*k*^)/2 *(the number of linear chromosomes) and L *= (*L*_*max *_+ 1)/2 *(the average number of genes in a duplicated segment). Then we approximate the expected vector for G*^*k*+1 ^*after one duplication operation with **where we have*

**Proof **In our model, we uniformly select a position to start duplicating *L *genes and transpose it to one new uniformly chosen position within the genome. The expected number of genes or adjacencies with exactly *i *copies within the duplicated segment is  or . The probability that the placement of the duplicated segment breaks one adjacency at any specific site is 1/(*N*^*k *^+ *l*^*k*^).

We again first ignore the adjacencies or telomeres in the original genome *G *created after a duplication event. Since we assume that no two genes or adjacencies are the same within the duplicated segment, we have

Now, we show that the correction for ignoring adjacencies or telomeres after a duplication event is  to each item . Consider any adjacency (*a*, *b*) in *G*: we might recover it if we move gene *a *next to gene *b *after the duplication. Since each gene has at most *C *copies in the genome, there are at most 2*LC*^2 ^possible duplication operations to recover that adjacency (*a*, *b*). There are altogether Ω (*L*(*N*^*k *^+ *l*^*k*^)^2^) different duplication operations. So, with probability , one specific adjacency in *G *might be created by the duplication event. Summing up all the *N *- *l *adjacencies in *G*, we see that the correction for ignoring the newly created adjacencies or telomeres in *G *is .

Similarly, we can get  and .

#### Gene loss

We uniformly select one gene with at least two copies and delete it.

**Theorem 3 ***Assume each gene has at most C *= *O*(1) *copies and **represents the current genome G*^*k *^*based on the original genome G. For conciseness, write **(the total number of genes) and l*^*k *^= (*ST*^*k*^+ *DT*^*k*^)/2 *(the number of linear chromosomes). Then we can write the expected vector for G*^*k*+1^*after one rearrangement operation as *, *where we have*

**Proof **In our model of gene loss, one gene with at least two copies is uniformly selected. The number of all possible genes to be deleted is . For  genes with exactly *i *copies in *G*^*k*^, the probability that one of them is selected and deleted is . So with probability , the number of genes with exactly *i *copies decreases by *i *and the number of genes with exactly (*i *-1) copies increases by (*i *-1).

We ignore the adjacencies or telomeres in the original genome *G *to be created after one gene loss. For  (*i *> 2) adjacencies with exactly *i *copies in *G*^*k *^which also appears in *G*, it is difficult to compute the number *f*_*i*_(*del*_*j*_) of such adjacencies that each single deletion *del*_*j *_(*j *= 1,..., *N*^*k *^- ) would affect. But we know that each adjacency with exactly *i *(*i *> 2) copies must relate to two genes with more than 2 copies, so we have . Considering *i *= 2,..., *C *and *C *= *O*(1), we have

For  adjacencies with exactly 1 copy in *G*^*k *^that also appears in *G*, it is also difficult to compute the number *f*_1_(*del*_*j*_) of such adjacencies that each single deletion *del*_*j *_(*j *= *N*^*k *^- ) would affect. Assume  is the count of genes with at least two copies but related to those adjacencies with exactly 1 copy in *G*^*k *^that also appear in *G*. We consider the effect of rearrangements, gene duplications and losses, and we approximate as follows:

For telomeres, we simply assume *ST *^*k*+1 ^= *ST *^*k *^and *DT *^*k*+1 ^= *DT *^*k*^.

Finally, we also approximate the number of adjacencies *RSA*^*k*+1 ^that we could thus ignore under rearrangements, gene duplications, and gene losses, and distribute it to the correction of  as follows:

Now, given *G*^0^, we estimate *E*(*V*_*G*_(*G*^*k*^)) for *k *> 0 by iterating *k *times the above formulas (using with *p*_*d *_and *p*_*l*_); at every step we identify *E*(*V*_*G*_(*G*^*k*-1^)) with the actual vector *V*_*G*_(*G*^*k*-1^).

**Corollary 1 ***The estimated vector **for all integers i *(0 ≤ *i ≤ k*) *can be computed in O*(*kC*) *time.*

## Results and discussion

We now present experimental results on the accuracy of our estimation of the expected vector after a given number of random evolutionary events and on the quality of our estimator for the true evolutionary distance (in terms of the actual number of evolutionary events). Our experiments all start with one genome with no duplicated genes and some chosen number of linear and circular chromosomes of various sizes. We first apply some number (usually 10) of duplication events (*L*_*max *_= 10 in all cases) to generate the original genome *G *with some initial duplicated genes. Then this genome is subjected to a prescribed number *k *of evolutionary events chosen according to *p*_*d *_and *p*_*l *_to obtain a final genome *G*^*k*^. We vary *k *from 0 to twice the number of genes. We ran tests on any types of initial genomes designed to resemble actual organismal genomes; we tested different choices of parameters on different genomes; and in each case we generated 10,000 runs to obtain a tight estimate of variance.

We compute the vector representations for all intermediate genomes and then use our method to estimate the evolutionary distance. Due to space limitations, we present results on just three initial genomes: 25,000 genes and 25 linear chromosomes (*p*_*d *_= 0.05, *p*_*l *_= 0.15); 10, 000 genes and 5 linear chromosomes (*p*_*d *_= 0.1, *p*_*l *_= 0.2); and 1, 000 genes and 1 circular chromosome (*p*_*d *_= 0.2, *p*_*l *_= 0.6). The first two examples match large and smaller metazoan genomes, the last matches a small bacterial genome.

### Accuracy of the expected vector after *k *random evolutionary events

**Figure 2:**
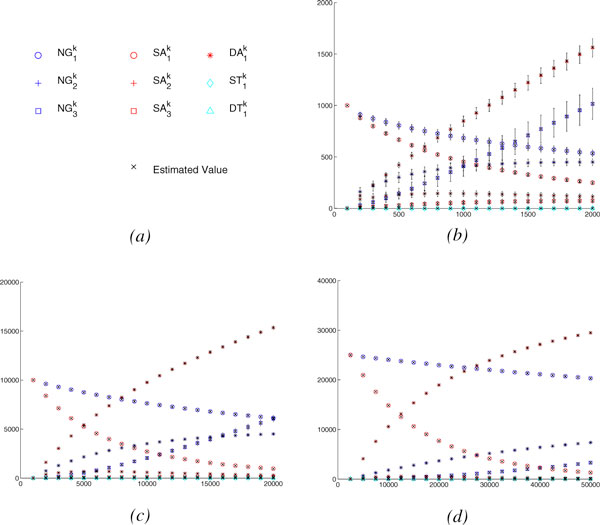
**The vector values as a function of the actual number of evolutionary events**. (*a*) the color and shape code for the values, (*b*) Gene # = 1,000, Linear Chromosome # = 0, Circular Chromosome # = 1, (*c*) Gene # = 10,000, Linear Chromosome # = 10, Circular Chromosome # = 0, (*d*) Gene # = 25,000, Linear Chromosome # = 25, Circular Chromosome # = 0.

We study the behavior of our estimator  by comparing its prediction to the sample mean for *V*_*G*_(*G*^*k*^), as computed from our 10,000 trials. In all of our experiments, we find that  is very close to the sample mean for *V*_*G*_(*G*^*k*^). Figure 2 shows the values in the vector as a function of the actual number of evolutionary events.  and  represent the number of adjacencies and genes with at least 3 copies in the original genome *G*, respectively. The figure shows that our estimation and the sample mean for *V*_*G*_(*G*^*k*^) are always very close.

### Accuracy of the estimation of the actual number of evolutionary events

We want to study the accuracy of our estimator for the actual number of evolutionary events; in order to do that, we create simulations with controlled numbers of evolutionary events and set up a threshold for correction in the estimation procedure. Specifically, we vary the actual number of evolutionary events from 0 to twice the number of genes in the original genome and we set 4 times the number of genes as an upper limit on the maximum number of evolutionary events. *C *is set to 10. Thus our estimated number *k *is chosen to minimize |  - *V*_*G*_(*F*)|_1_, the 1-norm distance between  and *V*_*G*_(*F*).

**Figure 3:**
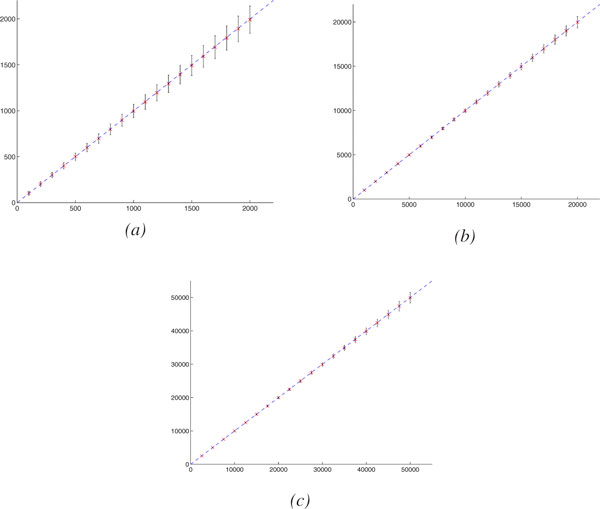
**The actual number of evolutionary events (*x *axis)vs. our estimator (*y *axis)**. (*a*) Gene # = 1000, Linear Chromosome # = 0, Circular Chromosome # = 1, (*b*) Gene # = 10000, Linear Chromosome # = 10, Circular Chromosome # = 0, (*c*) Gene # = 25000, Linear Chromosome # = 25, Circular Chromosome # = 0. Mean is indicated by × and standard deviation is indicated by vertical bar.

Figure 3 shows the mean and standard deviation for the actual number of evolutionary events estimated by our approach. Our approach provides accurate estimates, with very small variance.

**Figure 4:**
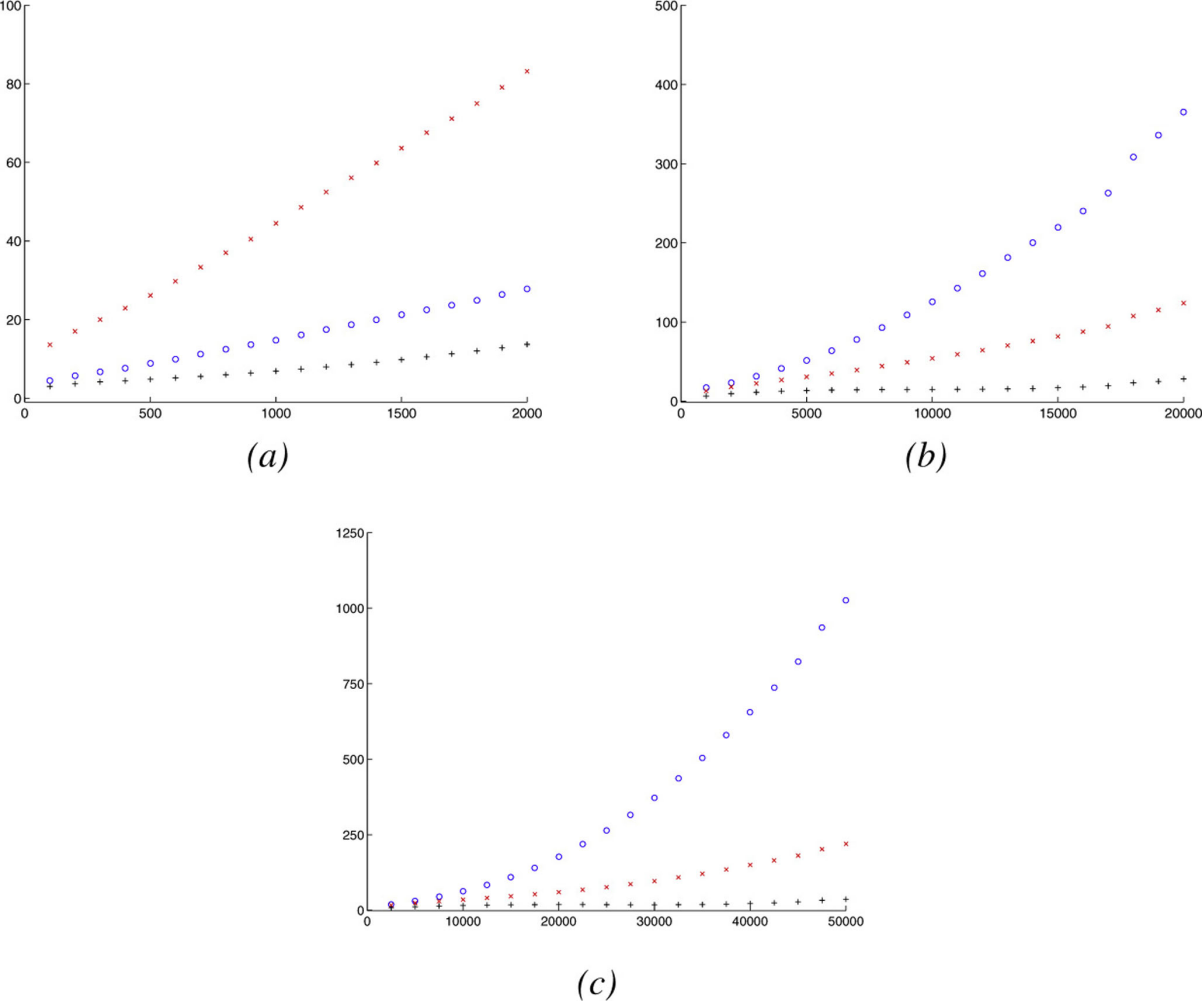
**The mean absolute difference between actual number of different evolutionary events and our estimation**. The actual number of evolutionary events (*x *axis) vs. The mean absolute difference between actual number of different evolutionary events and our estimation (*y *axis). (o: Rearrangements, +: Duplications, ×: Losses). (*a*) Gene # = 1000, Linear Chromosome # = 0, Circular Chromosome # = 1, (*b*) Gene # = 10000, Linear Chromosome # = 10, Circular Chromosome # = 0, (*c*) Gene # = 25000, Linear Chromosome # = 25, Circular Chromosome # = 0.

We also study the mean absolute difference between the actual number of evolutionary events and our estimator, shown in Figure 4.

Table 1 shows that the estimates are quite accurate up to very large numbers of events. Rearrangements, gene duplications, and gene losses fall under the category of "rare genomic events" (in the terminology of [14]), yet our estimator works well even for numbers that would instead indicate common events.

**Table 1:** Relative error of our estimator as a function of the actual number of evolutionary events

# genes	actual number of evolutionary events					
	# genes × 1			# genes × 2		
	
	Rearrangements	Duplications	Losses	Rearrangements	Duplications	Losses
1000	7.4%	3.4%	7.4%	6.9%	3.4%	6.9%
10,000	1.7%	1.4%	2.7%	2.6%	1.4%	3.1%
25,000	1.3%	1.5%	2.0%	2.6%	1.5%	2.9%

### Robustness to unknown model parameters

Up to now we have fixed *p*_*d *_and *p*_*l*_. We now consider the case in which these parameters are unknown--clearly the more common case in practice. We generate 10,000 cases with randomly chosen parameters *p*_*d *_and *p*_*l *_(at 1% resolution, *p*_*d *_< 4*p*_*l*_) and with actual numbers of evolutionary events varying from 0 to twice the number of genes, setting an upper limit of 4 times the number genes for the maximum number of evolutionary events.

Given the original genome, our estimated vector  is in fact a function of *i*, *p*_*d*_, and *p*_*l*_. We enumerate all possible values for *p*_*d *_and *p*_*l *_(at 1% resolution, *p*_*d *_< 4 *p*_*l*_). For each different pair of parameters *p*_*d *_and *p*_*l*_, we compute all  (*i *from 0 to 4 times the number of genes, *C *is set to 10). Our estimated number *k *is still chosen to minimize |  - *V*_*G*_(*F*)|_1_, the 1-norm distance between  and *V*_*G*_(*F*).

**Figure 5:**
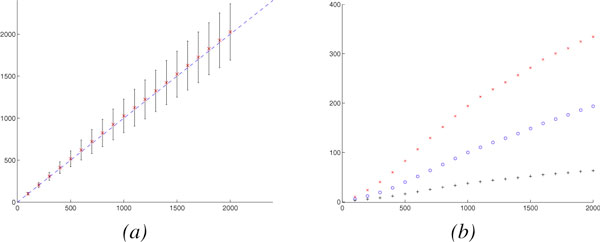
**The actual number of evolutionary events vs. our estimator for unknown model parameters**. (*a*): Mean (indicated by ×) and standard deviation (indicated by vertical bar) plots for the actual number of evolutionary events vs. our estimator (Gene # = 1000, Linear Chromosome # = 0, Circular Chromosome # = 1). (*b*): The mean absolute difference between actual number of different evolutionary events and our estimation (o: Rearrangements, +: Duplications, ×: Losses).

Figure 5 shows the comparison of our estimates to the actual number of evolutionary events. Our approach still provides accurate estimates in absence of known values for *p*_*d *_and *p*_*l *_and thus is quite robust. The mean absolute difference between the actual number of evolutionary events and our estimator becomes larger, especially when there are few common adjacencies left between the original and final genomes. (The duplications and losses may also partially cancel each other.)

## Conclusion

We propose a new evolutionary model for rearrangements, gene duplications and losses, and a corresponding method for estimating true evolutionary distance. The model is, to our knowledge, the first to preserve the structural dichotomy in genomic organization between most prokaryotes and most eukaryotes, and one of the few to unite rearrangements, duplications, and losses. Experimental results on a wide variety of genome structures exemplify the high accuracy and robustness of our estimator. This large gain in accuracy should translate into much better phylogenetic reconstructions as well as more accurate genomic alignments.

## Competing interest

The authors declare that they have no competing interests.

## Authors' contributions

YL conceived the idea, performed the analysis. YL, VR and KMS discussed and conducted the experiments. BMEM directed the project. YL and BMEM wrote the manuscript. All authors read and approved the final manuscript.
